# GDF10 is a negative regulator of vascular calcification

**DOI:** 10.1016/j.jbc.2024.107805

**Published:** 2024-09-21

**Authors:** Khrystyna Platko, Gabriel Gyulay, Paul F. Lebeau, Melissa E. MacDonald, Edward G. Lynn, Jae Hyun Byun, Suleiman A. Igdoura, Rachel M. Holden, Anna Roubtsova, Nabil G. Seidah, Joan C. Krepinsky, Richard C. Austin

**Affiliations:** 1 Department of Medicine, Division of Nephrology, McMaster University, and The Research Institute of St Joe’s Hamilton, Hamilton, Ontario, Canada; 2 Department of Biology, McMaster University Medical Centre, Hamilton, Ontario, Canada; 3 Department of Pathology and Molecular Medicine, Queen’s University, Kingston, Ontario, Canada; 4 Department of Medicine, Queen’s University, Kingston, Ontario, Canada; 5 The Institut de Recherches Cliniques de Montréal (IRCM), Affiliated with Université de Montréal, Montréal, Quebec, Canada

**Keywords:** cardiovascular disease, vascular smooth muscle cells, bone morphogenic protein (BMP), calcification, vascular biology

## Abstract

Cardiovascular mortality is particularly high and increasing in patients with chronic kidney disease, with vascular calcification (VC) as a major pathophysiologic feature. VC is a highly regulated biological process similar to bone formation involving osteogenic transdifferentiation of vascular smooth muscle cells (VSMCs). We have previously demonstrated that loss of T-cell death-associated gene 51 (TDAG51) expression leads to an attenuation of medial VC. We now show a significant induction of circulating levels of growth differentiation factor 10 (GDF10) in *TDAG51*^*−/−*^ mice, which was of interest due to its established role as an inhibitor of osteoblast differentiation. The objective of this study was to examine the role of GDF10 in the osteogenic transdifferentiation of VSMCs. Using primary mouse and human VSMCs, as well as *ex vivo* aortic ring cultures, we demonstrated that treatment with recombinant human (rh) GDF10 mitigated phosphate-mediated hydroxyapatite (HA) mineral deposition. Furthermore, *ex vivo* aortic rings from *GDF10*^−/−^ mice exhibited increased HA deposition compared to C57BL/6J controls. To explain our observations, we identified that rhGDF10 treatment reduced protein expression of runt-related transcription factor 2, a key driver of osteogenic transdifferentiation of VSMCs and VC. In support of these findings, *in vivo* treatment with rhGDF10 attenuated VD_3_-induced VC. Furthermore, we demonstrated an increase in circulating GDF10 in patients with chronic kidney disease with clinically defined severe VC, as assessed by coronary artery calcium score. Thus, our studies identify GDF10 as a novel inhibitor of mineral deposition and as such, may represent a potential novel biomarker and therapeutic target for the detection and management of VC.

Cardiovascular disease (CVD) is the leading cause of mortality in patients with chronic kidney disease (CKD) (1). Vascular calcification (VC) in the medial layer of the vessel wall due to hydroxyapatite (HA) mineral deposition, which subsequently leads to reduced vascular elasticity, is the predominant driver of this mortality (2). The prevalence of VC in patients with CKD increases from 40% in those with Stage 3 CKD to 80 to 90% in patients with Stage 5 CKD (3). Furthermore, VC is an independent risk factor for sudden cardiac death in patients with CKD (4, 5, 6). Despite the growing prevalence of CKD in the aging population (7), there is limited understanding of the molecular mechanisms that modulate the development of VC.

Although previously considered to be passive, VC is now recognized as a highly regulated pathological process that shares many features with bone formation (8). Bone morphogenic proteins (BMPs) are members of the transforming growth factor-*β* (TGF-*β*) superfamily that have long been shown to be crucial modulators of bone formation and are now recognized as drivers of VC (8, 9). Studies demonstrate that BMP2 and BMP4 are inducers of osteogenic differentiation and HA deposition in osteoblasts and vascular smooth muscle cells (VSMCs) (10, 11, 12). In contrast, BMP7 promotes a VSMC-specific phenotype by upregulating the expression of α-smooth muscle actin (α-SMA) (13). BMP3 is another negative regulator of osteoblast differentiation (14). Mice lacking the *BMP3* gene display increased bone mass (14), whereas transgenic mice overexpressing *BMP3* protein develop spontaneous rib fractures due to altered endochondral bone formation (15). BMP-3b, also known as growth differentiation factor 10 (GDF10), is a divergent member of the TGF-*β* superfamily that is closely related to BMP3 (16) and signals through TGF-*β* receptors (17, 18, 19). GDF10 expression correlates with osteoblast differentiation (20) and like BMP3, can inhibit this process (21). Supporting its role in osteogenic transdifferentiation, single-cell RNAseq of cells derived from the atherosclerotic aorta of Apolipoprotein E-deficient (Apoe^−/−^) mice revealed that GDF10 is involved in the ossification and osteoblast differentiation of VSMCs (22).

Runt-related transcription factor 2 (RUNX2) is a key transcription factor necessary for osteoblast development and maturation (23). Transcriptional regulation of RUNX2 by BMPs plays an important role in osteoblast differentiation, as well as mineralization of soft tissues (24, 25, 26, 27, 28, 29, 30). Combined treatment of fibroblast-like ligamentum flavum cells and primary skeletal myoblasts with adenoviruses to overexpress RUNX2 and BMP2 revealed a synergistic effect on the expression of osteogenic genes (30). Furthermore, overexpression of RUNX2 in VSMCs and adipose-derived stem cells promoted the expression of drivers of osteogenesis and a subsequent phenotypic switch (31, 32, 33). In VSMCs, phosphate treatment led to an increase in RUNX2 expression and activation (34). Additionally, an increase in RUNX2 expression was observed in calcified aortas (31, 35). Interestingly, Tandon and colleagues (36) demonstrated that there also exists an inverse relationship between RUNX2 and GDF10 expression in lung cancer cells. Likewise, mesenchymal stem cells from functionally deficient RUNX2 mice, characterized by impaired transcriptional activity due to compromised subnuclear targeting of RUNX2, also exhibit increased GDF10 expression (36, 37). RUNX2 overexpression and knockdown studies show that RUNX2 downregulates GDF10 expression by increasing its methylation status (36).

We have recently demonstrated that loss of T-cell death-associated gene 51 (TDAG51) expression reduced RUNX2 transcriptional activity and subsequently blocked the development of VC in TDAG51^−/−^ mice (34). Interestingly, our recent microarray analysis revealed that *GDF10* expression in cultured VSMCs derived from TDAG51^−/−^ mice was significantly upregulated compared to the VSMCs from littermate controls. To shed light on this critical observation, we now demonstrate that treatment with recombinant human (rh) GDF10 decreases phosphate (P_i_)-mediated RUNX2 expression and mineral deposition in VSMCs and *ex vivo* aortic ring cultures. Similarly, *in vivo* treatment with rhGDF10 attenuates VD_3_-induced VC. Furthermore, circulating GDF10 is increased in patients with CKD and clinically defined VC. Therefore, our studies identify GDF10 as a novel regulator of VC, serving as a potential therapeutic target for the management of VC and as a potential marker for its detection.

## Results

### TDAG51^−/−^ VSMCs and mice exhibit increased expression of GDF10, an inhibitor of osteogenesis

**Figure 1:**
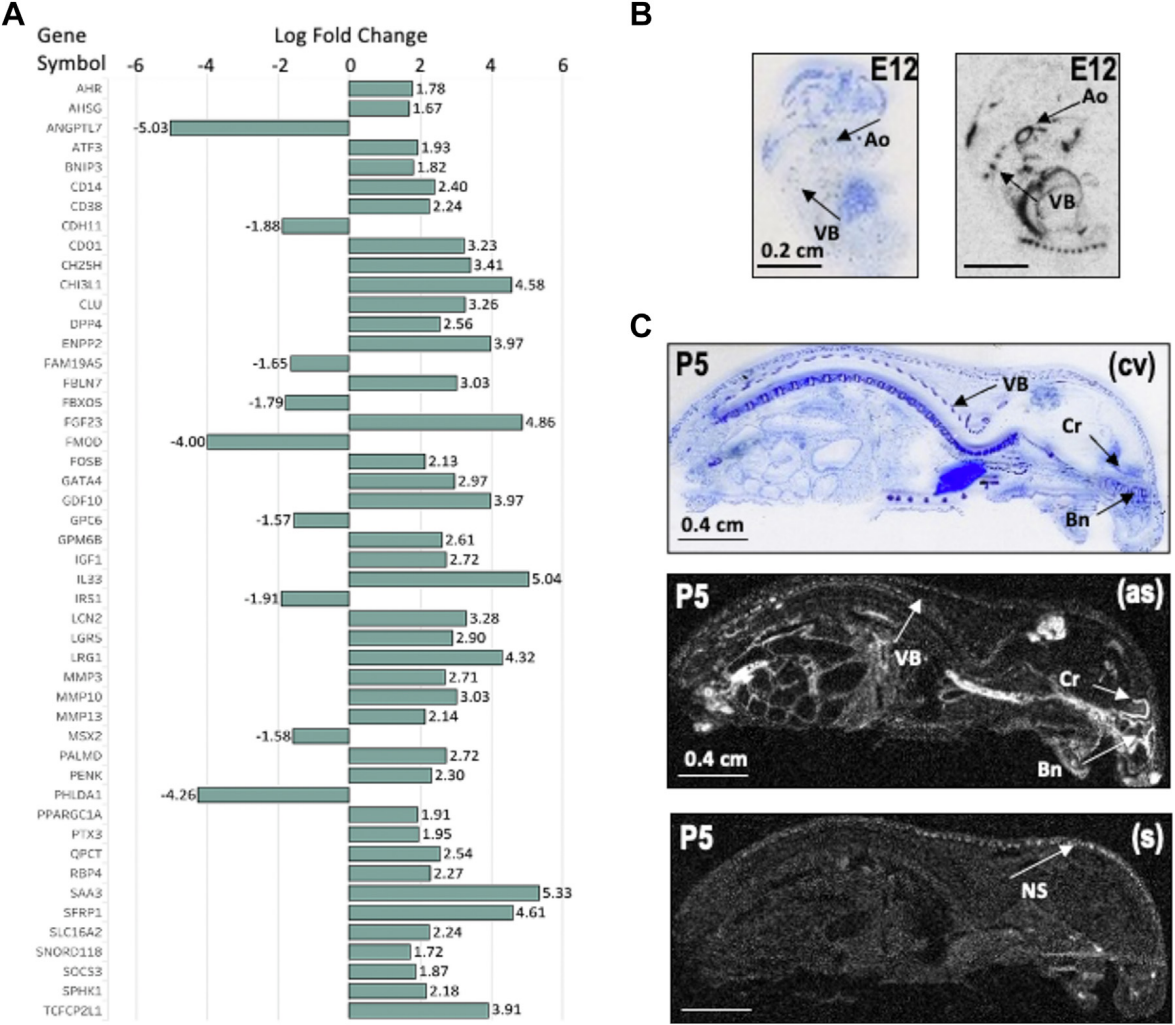
**GDF10 is upregulated in *TDAG51***^***−/−***^**mice and highly expressed in the vasculature, bone and cartilage.***A,* Representation of the log fold-change of genes that have previously been demonstrated to be involved in the regulation of mineral deposition in either soft tissues or bone in VSMCs from *TDAG51*^*−/−*^ mice compared to littermate controls. Data are shown as mean. *B* and *C*, *GDF10* mRNA distribution in whole body sections of mice at embryonic day 12 (E12) (B) and postnatal day 5 (p5) (C). As, antisense; S, control sense; CV, thionin staining; Ao, aorta; Bn, bone; Cr, cartilage; VB, vertebrae; NS, non-specific staining.

Osteogenic transdifferentiation of VSMCs is a major contributor to the development of VC associated with hyperphosphatemia (8). Our previous findings demonstrated that *TDAG51*^*−/−*^ VSMCs and mice exhibit an attenuation of HA deposition due to diminished RUNX2 activity (34). To further define additional modulators of VC, microarray analysis of *TDAG51*^*+/+*^ and *TDAG51*^*−/−*^ VSMCs identified 622 up-regulated and 363 down-regulated genes in *TDAG51*^*−/−*^ VSMCs. Identified genes were classified according to their molecular function, biological process, cellular component, and protein class (Fig. S1, *A*–*D*). Further analysis determined 49 genes, with log-fold change cut-off of ± 1.5 compared to *TDAG51*^*+/+*^ VSMCs, that have previously been demonstrated to be involved in the regulation of mineral deposition in either soft tissues or bone (Fig. 1*A*). Among the identified genes was *GDF10*, which was of interest because it is a circulating factor that has previously been shown to inhibit osteoblast differentiation, a process that draws many parallels with VC (8, 21). Consistent with the notion that GDF10 plays a role in bone formation and vascular mineral deposition (20, 21), *in situ* hybridization studies reveal that at embryonic day 12 (E12) and post-natal day 5 (p5), *GDF10* mRNA was abundantly expressed in the vasculature such as the aorta, as well as in bone, including the vertebrae, skull and cartilage (Fig. 1, *B* and *C* and S2, *A*–*C*).

**Figure 2:**
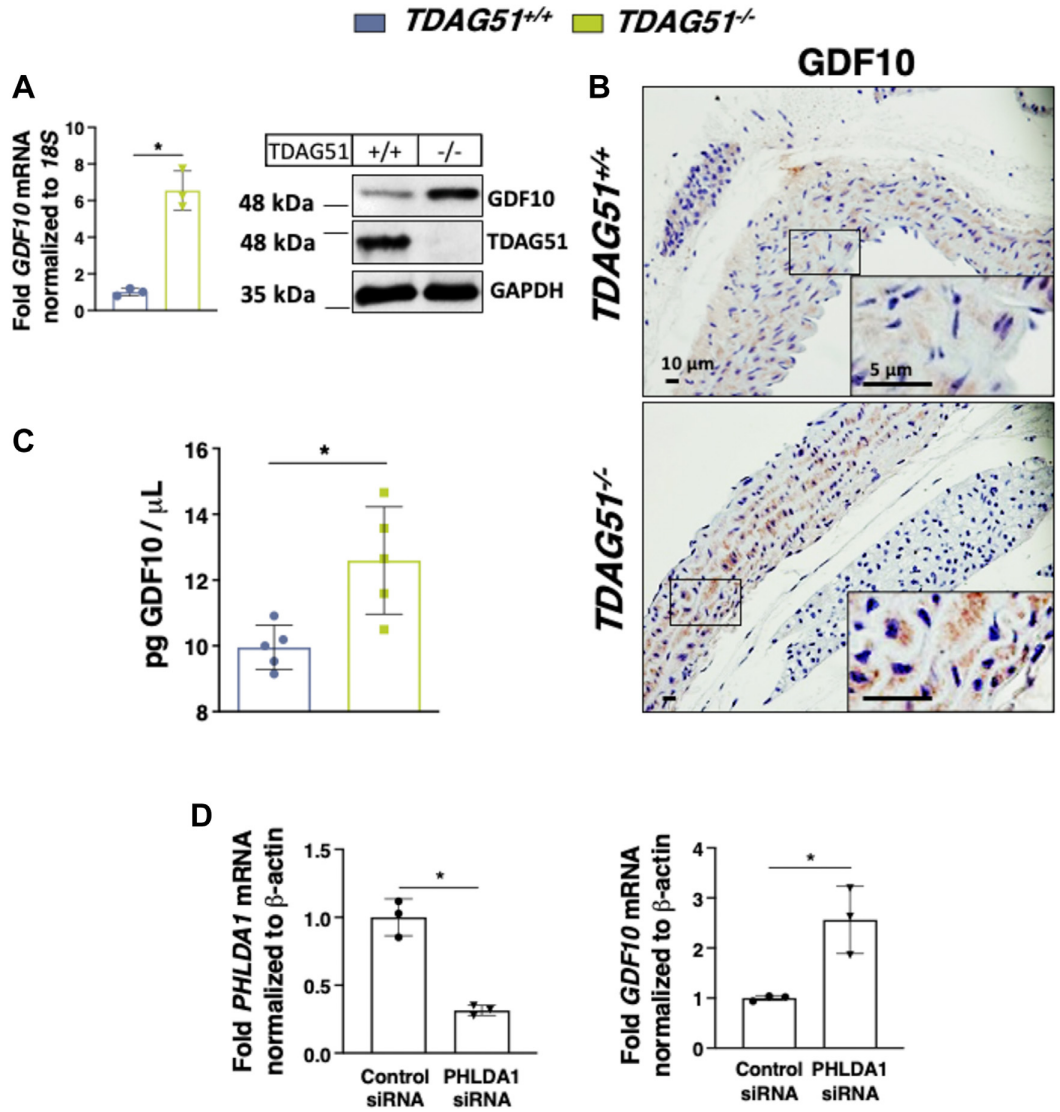
***TDAG51***^***−/−***^**VSMCs and mice exhibit increased expression of GDF10.***A*, quantitative real-time PCR and immunoblot analysis of GDF10 expression in *TDAG51*^*+/+*^ and *TDAG51*^*−/−*^ VSMCs (n = 3). *B*, IHC staining of GDF10 in *TDAG51*^*+/+*^ and *TDAG51*^*−/−*^ mouse aortas. *C*, plasma levels of GDF10 protein in male *TDAG51*^*+/+*^ and *TDAG51*^*−/−*^ mice (n = 5). *D*, quantitative real-time PCR analysis of *PHLDA1* and *GDF10* expression in human VSMCs transfected with either control siRNA or siRNA targeted against *PHLDA1* (n = 3). All data are shown as mean and error bars as SD. ∗*p* < 0.05 by 2-tailed unpaired Student *t* test. ∗, *p* < 0.05 vs. control as indicated in the figure for each applicable figure panel.

Consistent with the microarray data, quantitative real-time PCR and immunoblot analysis demonstrated an increase in *GDF10* mRNA and protein expression in *TDAG51*^*−/−*^ VSMCs (Fig. 2*A*). Similarly, immunohistochemistry (IHC) detection of GDF10 (Fig. 2*B*) as well as circulating levels of GDF10 (Fig. 2*C*) were increased in *TDAG51*^*−/−*^ mice compared to controls. In support of these findings, siRNA-mediated knockdown of *PHLDA1* (human homolog of TDAG51) in human VSMCs resulted in increased *GDF10* expression (Fig. 2*D*), thus suggesting that TDAG51 negatively regulates GDF10 expression. Given that loss of TDAG51 leads to (i) elevated expression of GDF10 as well as (ii) an attenuation of HA deposition, our findings suggest that similar to its role in osteoblast differentiation, GDF10 likely represents a circulating inhibitor of mineral deposition in VSMCs.

### Recombinant human GDF10 treatment attenuates P_i_-mediated mineral deposition

**Figure 3:**
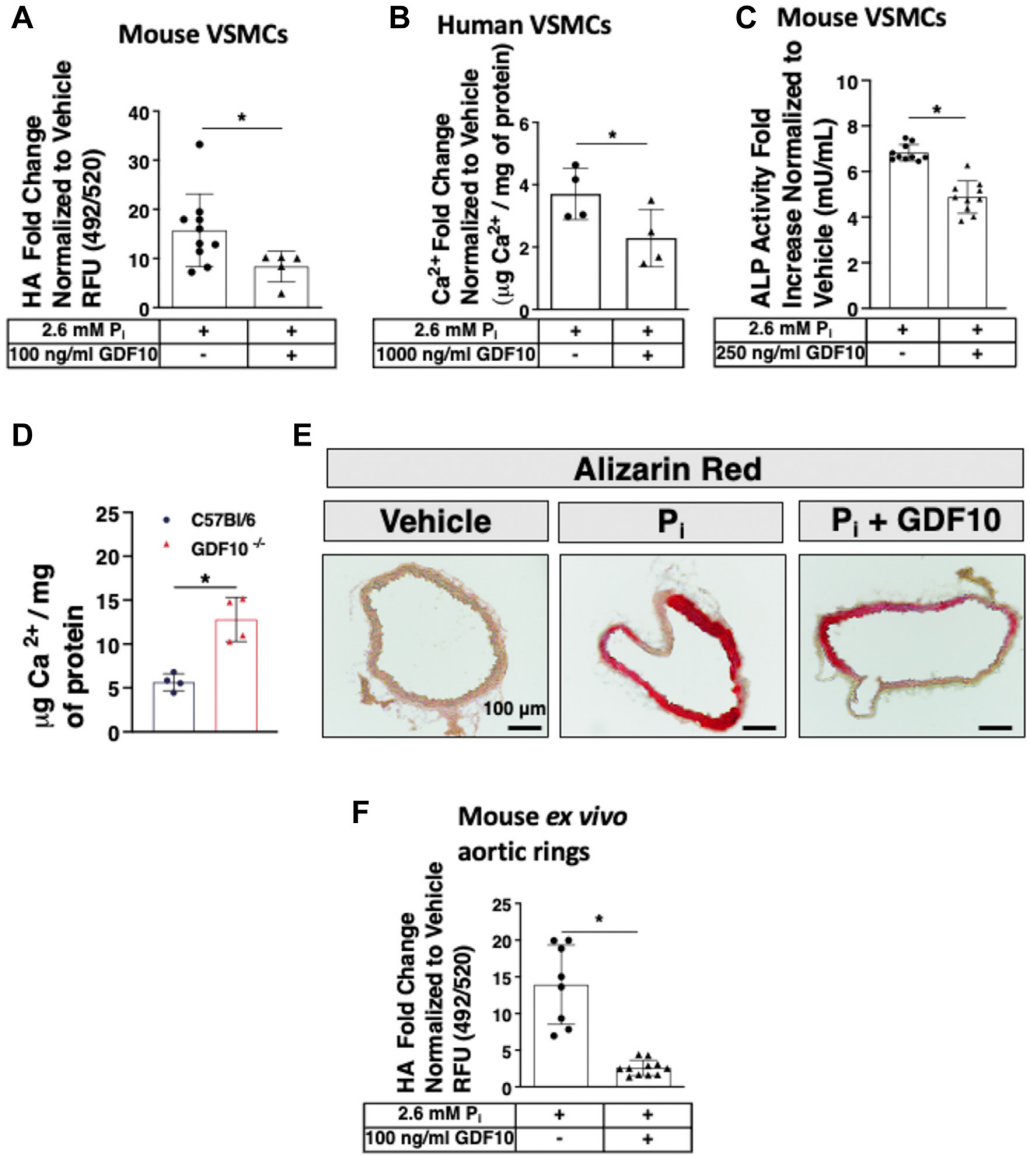
**rhGDF10 treatment inhibits P**_**i**_**-mediated mineral deposition.***A*, quantification of P_i_-induced HA crystal formation in wild-type (C57BL/6J) mouse VSMCs (n = 5-10) following P_i_ (2.6 mM) treatment for 5 in the presence or absence of rhGDF10 (100 ng/ml). *B,* Quantification of P_i_-induced HA crystal formation in human VSMCs following 5 days of P_i_ (2.6 mM) treatment in the presence or absence of rhGDF10 (100 ng/ml; n = 8-11). *C*, quantification of alkaline phosphatase activity in wild-type VSMCs following 4 days of P_i_ (2.6 mM) and rhGDF10 (250 ng/ml) treatment. *D*, Quantification of calcium deposition in C57BL/6J and *GDF10*^*−/−*^ mouse *ex vivo* aortic ring cultures following 7 days of P_i_ (2.6 mM) treatment (n = 4). *E*, Alizarin Red calcium stain in *ex vivo* aortic rings following 7 days of P_i_ (2.6 mM) and rhGDF10 (1000 ng/ml) treatment. *F*, quantification of P_i_-induced HA crystal formation in wild-type (C57BL/6J) mouse *ex vivo* aortic ring cultures (n = 4) following P_i_ (2.6 mM) treatment for 7 days, in the presence or absence of rhGDF10 (1000 ng/ml). All data are shown as mean and error bars as SD. ∗*p* < 0.05 by 2-tailed unpaired Student *t* test. ∗, *p* < 0.05 vs. control as indicated in the figure for each applicable figure panel.

Given that GDF10 signals through TGF-*β* receptors (17, 18, 19), we first confirmed that treatment with rhGDF10 induces SMAD3 phosphorylation in a TGF-*β* receptor-dependent manner (Fig. S3*A*). We next examined the effect of rhGDF10 on mineral deposition in primary mouse VSMCs and *ex vivo* aortic ring cultures, as well as primary human VSMCs. Consistent with its previously established role as an inhibitor of osteoblast differentiation (21), rhGDF10 attenuated P_i_-mediated mineral deposition in primary mouse (Fig. 3*A*) and human VSMCs (Fig. 3*B*). Similarly, rhGDF10 reduced Pi-induced alkaline phosphatase (ALP) activity (Fig. 3*C*). *Ex vivo* aortic rings from *GDF10*^*−/−*^ mice exhibited increased mineral deposition compared to those from *C57BL/6J* controls in high P_i_ (Fig. 3*D*). The effect of rhGDF10 treatment on the pattern of mineral accumulation in *ex vivo* aortic rings (Fig. 3*E*) are consistent with the mineral deposition results. Furthermore, rhGDF10 attenuated P_i_-mediated mineral deposition in *ex vivo* aortic ring cultures (Fig. 3*F*). Collectively, these data indicate that GDF10 is a negative regulator of P_i_-mediated mineral deposition in VSMCs.

Similar to other TGF-*β* family members, active GDF10 is generated by proteolytic cleavage of the precursor protein at the putative furin cleavage site (RXXR) (38, 39, 40). In order to confirm that active GDF10 is necessary for the observed anti-osteogenic phenotype, human and mouse VSMCs were treated with either cleaved/active (Gln369-Arg478) or full-length (FL) rhGDF10 in the presence or absence of an established furin inhibitor, decanoyl-RVKR-CMK (Fig. S3, *B* and *C*) (41). Both FL and active peptide forms of rhGDF10 significantly reduced calcification, while decanoyl blocked the inhibitory effect of the FL rhGDF10. A scrambled peptide was also used as an additional control and had no effect on HA deposition. Thus, our findings suggest that only active GDF10 acts as an inhibitor of HA deposition and VC. Given that decanoyl-RVKR-CMK inhibited the anti-calcific effect of GDF10, these observations also suggest that GDF10 is processed by furin.

### Recombinant human GDF10 treatment inhibits RUNX2 expression and activity

**Figure 4:**
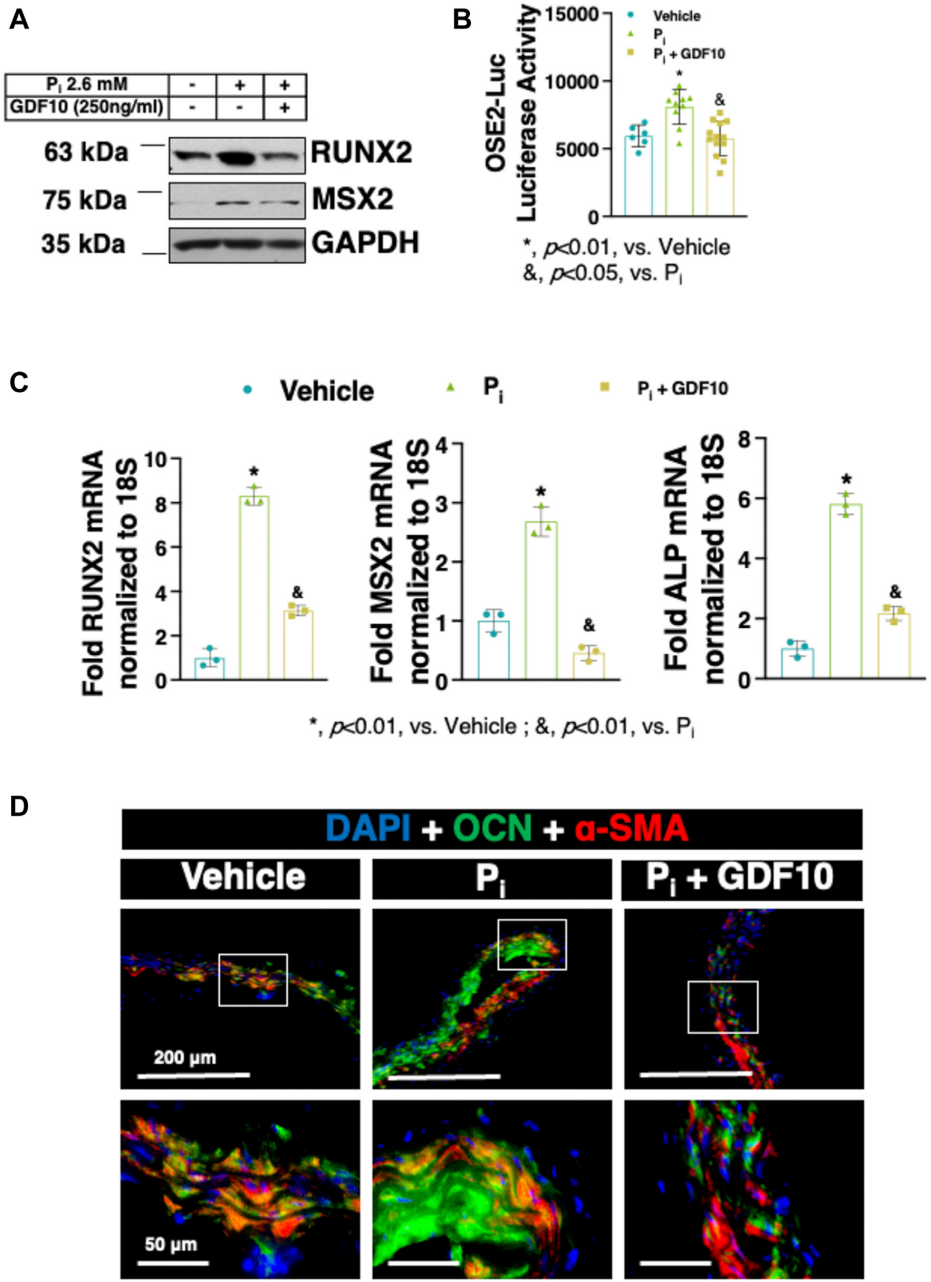
**rhGDF10 treatment inhibits P**_**i**_**-induced RUNX2 protein expression in C57BL/6J VSMCs.***A*, Immunoblot analysis of RUNX2, MSX2, and GAPDH expression in VSMCs from C57BL/6J mice following 4-days treatment of P_i_ (2.6 mM) in the presence or absence rhGDF10 (250 ng/ml). *B*, RUNX2 luciferase reporter assay following 2 days of 2.6 mM P_i_ treatment ± rhGDF10 (n = 6-13 per group). *C*, quantitative real-time polymerase chain reaction of RUNX2, MSX2, and ALP mRNA expression in VSMC treated as in (B) (n = 3). *D*, representative micrograph of immunofluorescence staining in wild-type *ex vivo* aortic rings following 7 days of P_i_ (2.6 mM) and rhGDF10 (1000 ng/ml) treatment, OCN (*green*), α-SMA (*red*) and DAPI (*blue*). All data are shown as mean and error bars as SD. ∗, *p* < 0.01, vs. Vehicle; &, *p* < 0.01, vs. P_i_ by 1-way ANOVA with Tukey multiple comparison testing using Prism 6 (GraphPad) as indicated in the figure for each applicable figure panel.

Osteogenic transdifferentiation of the medial layer of the vessel wall leading to VC draws many similarities to the growth and maturation of osteoblasts (8). We next examined the effect of rhGDF10 on the expression of well-established drivers of osteogenic transdifferention of VSMCs (26, 27, 33). Immunoblot analysis demonstrated that rhGDF10 attenuated P_i_-mediated upregulation of RUNX2 and its transcriptional cofactor Msh homeobox 2 (MSX2) (Fig. 4*A*). Because the expression of RUNX2 and MSX2 is associated with RUNX2-mediated transcriptional activity and a pro-osteogenic phenotype (26, 42), RUNX2 transcriptional activation was also examined using 6XOSE2-Luc. Fig 4*B* shows that P_i_-induced RUNX2 activation was inhibited by rhGDF10. In line with these findings, the mRNA expression of established markers of osteogenesis, including RUNX2, MSX2, and ALP was also reduced upon rhGDF10 treatment (Fig. 4*C*). Additionally, rhGDF10 treatment decreased expression of the RUNX2 target osteocalcin (OCN) in *ex vivo* aortic rings from C57BL/6J mice following 7 days of treatment (Fig. 4*D*). Thus, these observations reveal that GDF10 may in part inhibit arterial mineral deposition by regulating the expression of key drivers of VC, such as RUNX2 and its downstream targets.

### Murine ablation of GDF10 expression has no effect on VC

**Figure 5:**
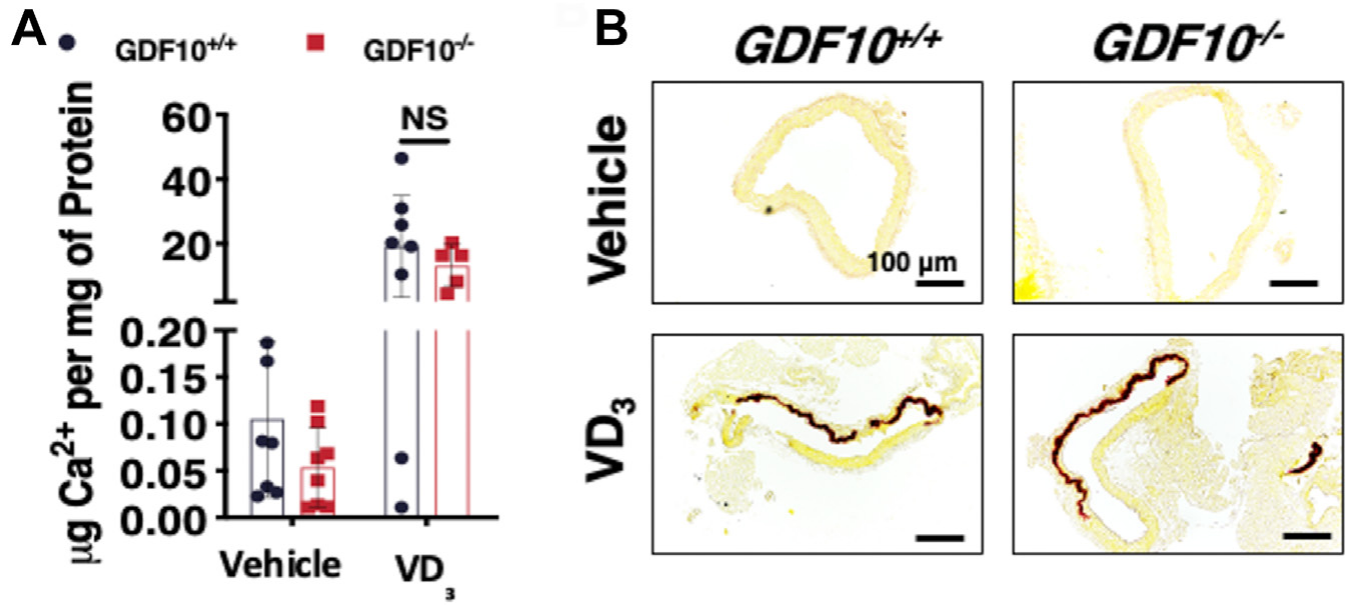
**GDF10**^**−/−**^**mice develop a similar degree of VD**_**3**_**-induced mineral deposition compared to GDF10**^**+/+**^**littermate controls.***A* and *B*, Calcium quantification and Alizarin Red calcium stain in the abdominal aortas (n = 5-8). All data are shown as mean and error bars as SD, with no statistical significance (NS) by 1-way ANOVA with Tukey multiple comparison testing using Prism 6 (GraphPad) as indicated in the figure for each applicable figure panel.

To investigate the role of GDF10 in the development of VC *in vivo*, a model of vitamin D3 (VD_3_)-induced VC was employed. Mineral deposition within the vessel wall was examined by quantification of Ca^2+^ content (Fig. 5*A*) and Alizarin Red calcium stain (Fig. 5*B*). To our surprise, no difference in mineral deposition was observed between *GDF10*^*−/−*^ and *GDF10*^*+/+*^ littermate controls.

### rhBMP3 inhibits VC *in vitro* and is elevated in the circulation of GDF10^−/−^ mice with VC

**Figure 6:**
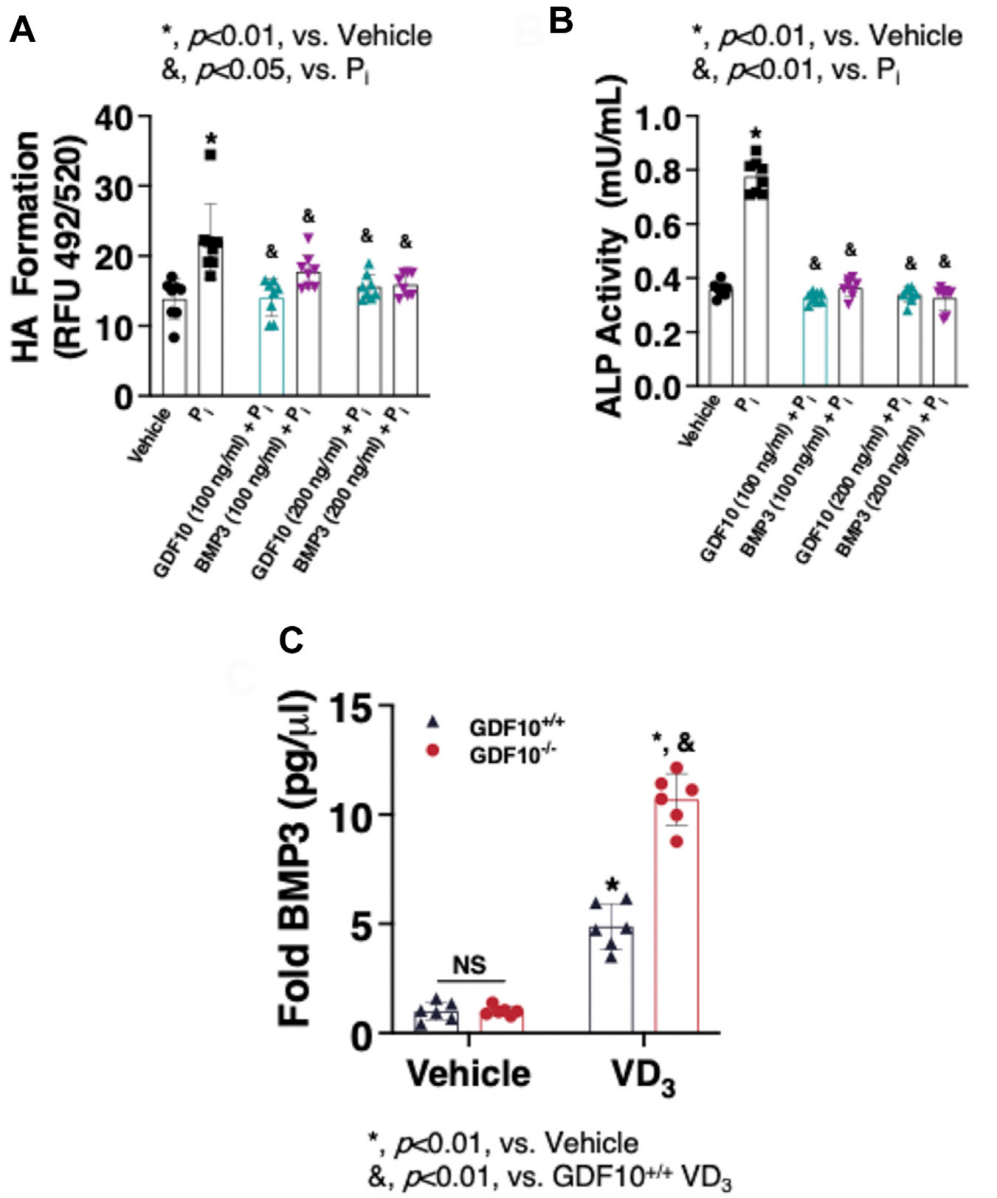
**rhBMP3 inhibits VC *in vitro* and is elevated in the circulation of GDF10**^**−/−**^**mice with VC.***A*, HA crystal formation in wild-type (C57BL/6J) mouse VSMCs (n = 5-10) following P_i_ (2.6 mM) treatment for 5 days in the presence or absence of rhGDF10 (100-200 ng/ml; n = 8) or BMP3 (100-200 ng/ml; n = 8). *B*, Quantification of alkaline phosphatase activity in wild-type VSMCs following 4 days of P_i_ (2.6 mM) and rhGDF10 rhGDF10 (100-200 ng/ml; n = 8) or BMP3 (100-200 ng/ml; n = 8). *C*, circulating BMP3 levels in GDF10^+/+^ or GDF10^−/−^ (n = 6) as measured by ELISA. All data are shown as mean and error bars as SD. ∗, *p* < 0.01, vs. Vehicle; &, *p* < 0.01/0/05, vs. P_i_/GDF10^+/+^ VD_3_, by 1-way ANOVA with Tukey multiple comparison testing using Prism 6 (GraphPad) as indicated in the figure for each applicable figure panel.

BMP3 is another member of the TGF-*β* family which has previously been demonstrated to be a powerful negative modulator of osteogenic differentiation and mineralization in mouse myoblast cells (14). Interestingly, there exists a high degree of structural similarity between GDF10 and BMP3 (16). Furthermore, there also appear to be functional similarities between GDF10 and BMP3, although these were reported across different disease models. We thus compared the effect of rhGDF10 and rhBPM3 on mineral deposition in mouse VSMCs. Similar to rhGDF10, rhBPM3 attenuated mineral deposition (Fig 6*A*) and ALP activity (Fig. 6*B*) in mouse VSMCs. To interrogate the possibility of a compensatory increase in BMP3 (14, 15), circulating levels were measured using ELISAs (Fig. 6*C*). These data revealed a substantial increase in circulating BMP3 in *GDF10*^*−/−*^ mice, likely compensating for the absence of GDF10 and potentially explaining the inconsistency between our *in vivo* and *ex vivo* observations.

### Recombinant human GDF10 treatment inhibits VC *in vivo*

**Figure 7:**
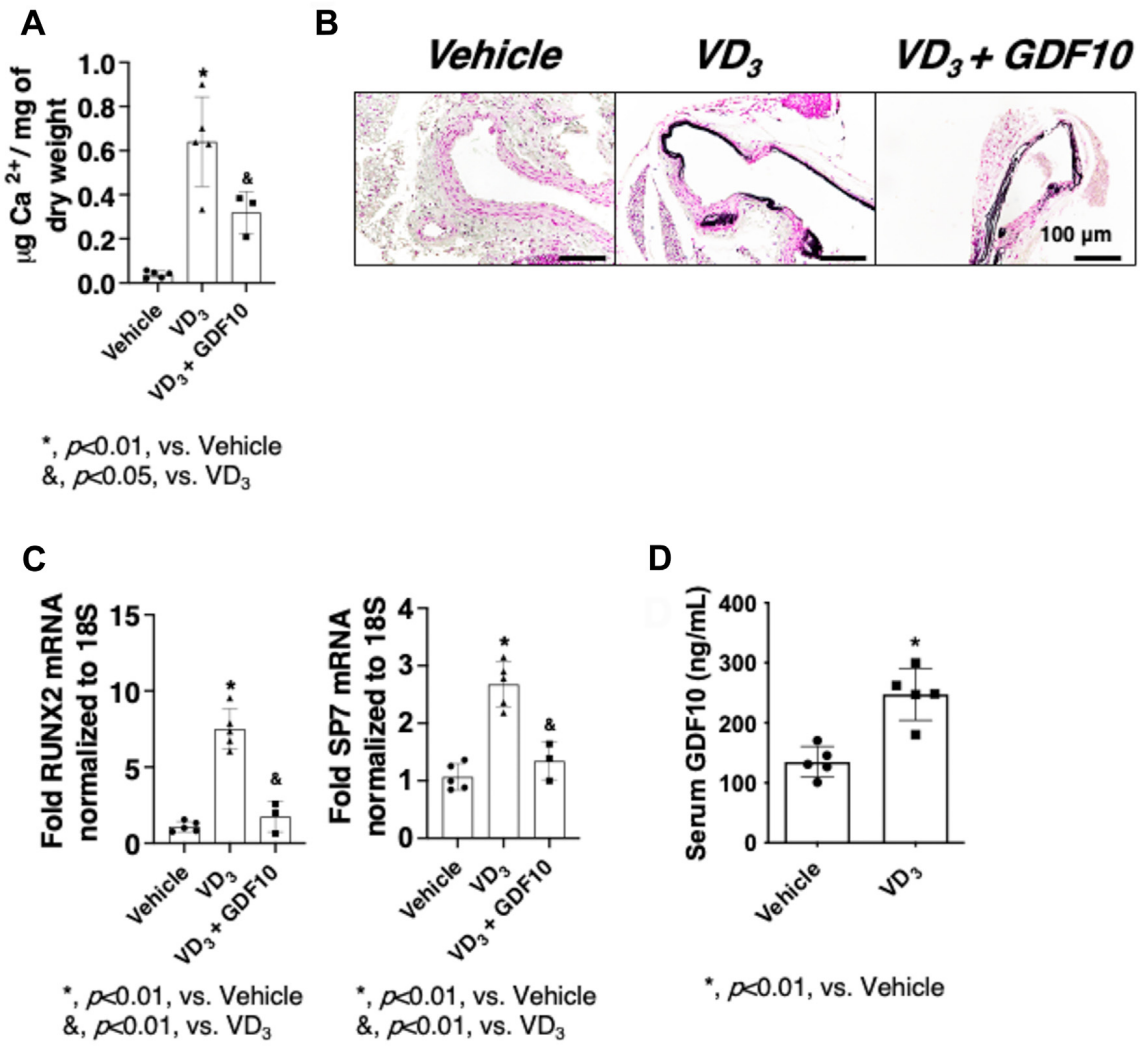
**rhGDF10 treatment inhibits VC *in vivo*.***A*, calcium quantification in the abdominal aortas (n = 3-5) from C57BL/6J mice following P_i_ VD3 treatment for 4 days in the presence or absence of rhGDF10. *B*, Von Kossa stain of the abdominal aortas (n = 3-5). *C*, Quantitative real-time polymerase chain reaction of RUNX2 and SP7 mRNA expression in the abdominal aortas (n = 3-5) from C57BL/6J mice following P_i_ VD3 treatment for 4 days in the presence or absence of rhGDF10. All data are shown as mean and error bars as SD. ∗, *p* < 0.01, vs. Vehicle; &, *p* < 0.01/0.05, vs. VD_3_, by 1-way ANOVA with Tukey multiple comparison testing using Prism 6 (GraphPad) as indicated in the figure for each applicable figure panel. *D*, serum GDF10 levels in mice treated with or without VD3 for 4 days.

To further interrogate the effect of GDF10 on VC *in vivo*, we tested the effects of rhGDF10 on VD_3_-mediated VC in mice. Mineral deposition within the vessel wall, examined by quantification of Ca^2+^ content, was attenuated by rhGDF10 (Fig 7*A*). Consistent with the quantification of mineral deposition, Von Kossa staining of arteries from mice treated with VD_3_ and rhGDF10 show a modest reduction in mineral deposition in the medial layer of the vessel wall. Importantly, arteries from mice treated with rhGDF10 show a more preserved integrity of the vessel wall structure compared to the VD3 group (Fig. 7*B*). Additionally, mRNA expression of known markers of osteogenesis, RUNX2, and SP7, was also reduced in the arteries of mice treated with rhGDF10 (Fig. 7*C*). In conclusion, these observations are in line with our *in vitro* data and suggest that GDF10 is a negative regulator of osteogenesis.

Consistent with the role of BMPs as response to injury factors (8, 43, 44, 45), we also observed an increase in circulating GDF10 in WT mice treated with recombinant VD_3_. This suggests that elevated levels of GDF10 serve to combat the progression of mineral deposition in mice (Fig. 7*D*).

### Circulating GDF10 levels are elevated in patients with coronary artery calcification

**Figure 8:**
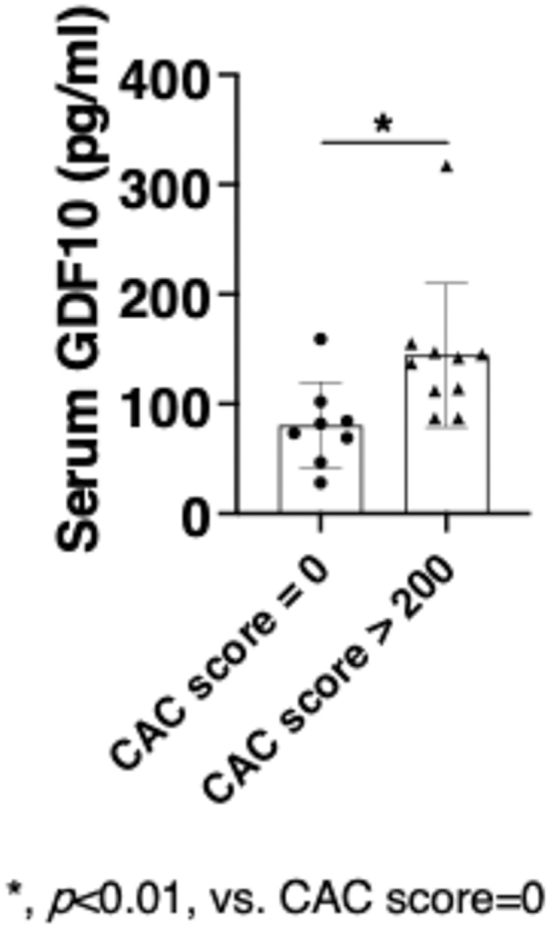
**Serum GDF10 levels are elevated in patients with CKD and established VC.***A*, quantification of serum GDF10 levels in patients with CKD (n = 8-9). All data are shown as mean and error bars as SD. ∗, *p* < 0.01, vs. CAC score=0 by 2-tailed unpaired Student *t* test as indicated in the figure for each applicable figure panel.

Given the established role of circulating osteogenic proteins in the development of arterial calcification (43, 44, 45), we next examined circulating GDF10 levels in a cohort of 18 patients with CKD having severe VC, as assessed by coronary artery calcification (CAC) score of >200 (Fig. 8). Additional details about the patient population are summarized in Table 1. Similar to other established inhibitors of medial arterial mineral deposition (8, 43, 44, 45), we observed an increase in serum GDF10 in patients with severe VC, suggesting a compensatory increase of this response to injury factor in order to counteract the progression of VC.

**Table 1:** Patient information

Parameter	Not stratified Mean (S.D) (n = 18)	CAC = 0 Mean (S.D.) (n = 7)	CAC > 200 Mean (S.D.) (n = 11)
Age (years)	61.18 (14.40)	51.78 (8.89)	67.90 (13.92)
GFR (mL/min)	29.65 (11.33)	35.19 (10.05)	25.78 (10.96)
Calcium (mmol/L)	2.41 (0.22)	2.43 (0.07)	2.40 (0.29)
Phosphate (mmol/L)	1.17 (0.22)	1.20 (0.27)	1.15 (0.19)^a^
CAC (Agatston units)	627.59 (809.92)	N/A	N/A

a *P* < 0.05 *versus* CAC = 0.

## Discussion

The relationship between TGF-*β* signaling and VC has been known for decades and has predominantly focused on the effect of well-characterized members of this diverse superfamily, such as TGF-*β*, BMP2, and BMP7 (24, 25, 26, 27, 28, 30). Our studies begin to characterize the relationship between a divergent member of the TGF-*β* superfamily, GDF10, and the osteogenic transdifferentiation of VSMCs. Using a combination of *in vitro, ex vivo,* and *in vivo* studies, we demonstrated that rhGDF10 attenuated VC. To explain these observations, we also identified that rhGDF10 reduced the expression of key drivers of osteogenic transdifferentiation in VSMCs.

Osteogenic transdifferentiation of VSMCs is a highly regulated process that shares many similarities with osteoblast differentiation and bone mineralization (8). RUNX2 is an indispensable transcription factor necessary for both of these processes to occur (46, 47). Studies demonstrate that RUNX2 is essential for osteogenic transdifferentiation of VSMCs and adipose-derived mesenchymal stem cells (32, 47). Importantly, accumulating evidence demonstrates that several members of the BMP family are key regulators of RUNX2 transcriptional activity and expression in the context of osteoblast differentiation, as well as mineralization of soft tissues (27, 28, 29, 30, 48). In osteoblasts, TGF-β inhibits differentiation through Smad3-mediated repression of Runx2 function (49). In human aortic smooth muscle cells, site-directed mutagenesis analysis indicated that transcriptional repression of Runx2 was mediated by its direct interaction with SMAD3 (50). Given that GDF10 exerts its effect *via* the TGF-β1/SMAD3 pathway in several disease models (17, 18, 51), it is likely that GDF10 modulates RUNX2 *via* the same axis.

We have recently demonstrated that VSMCs derived from *TDAG51*^*−/−*^ mice exhibit reduced expression and transcriptional activity of RUNX2, leading to diminished VC (34). Here we illustrate that reduced VC in *TDAG51*^*−/−*^ mice is also accompanied by an increase in expression and circulating levels of *GDF10*. Evidence also suggests that there exists an inverse relationship whereby RUNX2 can regulate GDF10 expression in lung carcinoma and mesenchymal stem cells (36). Furthermore, GDF10 blocks osteoblast differentiation by attenuating the expression of RUNX2, OCN, and Collagen 1 (21). These findings provide an additional mechanism to explain the protective phenotype observed in *TDAG51*^*−/−*^ mice (34) In the present study, we provide direct evidence that rhGDF10 treatment inhibits P_i_-mediate HA mineral deposition in cultured mice and human VSMCs, as well as *ex vivo* aortic ring cultures. Additionally, rhGDF10 treatment attenuated RUNX2 expression and transcriptional activity in wild-type VSMCs and protected against VC *in vivo*. Thus, our findings support the role of GDF10 as a novel negative modulator of RUNX2 and VC.

GDF10 is a member of the TGF-*β* superfamily that also shares 83% amino acid sequence homology with BMP3 (16). Daluiski and colleagues have demonstrated that BMP3 is a powerful negative modulator of osteogenic differentiation and mineralization in mouse myoblast cells. However, no skeletal defects were observed in *BMP3*^*−/−*^ embryos and newborns. Additionally, the authors observed no obvious skeletal phenotype in adult *BMP3*^*−/−*^ mice other than an increase in total trabecular bone volume, which was accompanied by a considerable amount of variation between mice (14). Consistent with the role of BMP3 as an inhibitor of osteogenesis, transgenic mice overexpressing *BMP3* develop spontaneous rib fractures due to defects in bone formation (15). Due to the homology between these two proteins, in a manner similar to BMP3, GDF10 expression also correlates with periods of osteoblast differentiation in rat calvarial osteoblasts (20, 21). To our surprise, however, we observed no difference in HA mineral deposition between VD_3_-treated *GDF10*^*−/−*^ and *GDF10*^*+/+*^ mice. Interestingly, the circulating levels of BMP3 were elevated in *GDF10*^*−/−*^ mice. Thus, similar to the studies conducted in *BMP3*^*−/−*^ mice (14), the absence of a VC phenotype observed in *GDF10*^*−/−*^ mice from our study may be due to its functional redundancy with its closely related family member, BMP3.

Our understanding of BMPs is expanding beyond that of bone growth and cartilage formation. Several studies have now demonstrated that BMPs act as *response to injury* factors, thereby orchestrating tissue regeneration, repair, and mitigating disease progression (18, 19, 52, 53, 54, 55, 56). These signaling proteins contribute to the activation of cellular pathways that promote not only bone formation but also the healing of various soft tissues. Clinical and pre-clinical studies demonstrate an increase in circulating BMP9 levels in patients with liver fibrosis as well as an increase in perivascular hepatic fibrosis in *BMP9* knockout mice (52, 53). Exposure of primary liver sinusoidal endothelial cells to exogenous BMP9 led to an attenuation of the fibrotic response (53). In a similar manner, BMP7 treatment can prevent tubulointerstitial fibrosis and preserve renal function in a rodent model of renal injury (55, 56). Consistent with these findings, exogenous BMP7 also ameliorates the expression of profibrotic genes in the aortas of uremic rats (54). On the other hand, *GDF15* expression dramatically increases following liver injury (57), while its overexpression ameliorates ischemia-reperfusion injury (58) and leads to improved functional recovery following a traumatic spinal cord injury (59). Recent evidence also suggests that GDF10 plays a role in injury prevention and wound healing, as Li and colleagues (19) demonstrate an increase in GDF10 expression in rodent and non-human primate models of stroke, as well as in autopsy samples from patients who presented with a clinically diagnosed stroke event. Furthermore, exogenous treatment with rhGDF10 promoted axonal sprouting and functional recovery after stroke in rodents (19). Treatment with recombinant GDF10 was demonstrated to attenuate TGF-*β*-induced fibrosis of lung fibroblasts (60). Similarly, the administration of GDF10 in aged mice reversed sarcopenia; pointing to the importance of GDF10 in muscle repair (61). In line with these findings, GDF10-null fibroblasts were more susceptible to TGF-*β*-induced fibrogenic changes, while GDF10-null mice exhibited exaggerated lung fibrosis induced by silica-nanoparticles *in vivo* (60). We and others also demonstrated that *GDF10*^*−/−*^ mice develop severe hepatic fibrosis and lipid accumulation, whereas mice with adipose-specific overexpression of BMP-3b were protected against diet-induced obesity (18, 62, 63). In our present study, we demonstrate an increase in serum GDF10 in CKD patients with established VC. Similar to other BMP family members, GDF10 may potentially act as an inhibitor of VC and a circulating *response to injury* protective factor.

The present study identifies GDF10 as a novel mediator of VSMC osteogenic transdifferentiation and HA mineral deposition. Mechanistically, rhGDF10 treatment inhibited P_i_-induced RUNX2 expression and activity. Our results also show that patients with CKD and established severe VC exhibit elevated circulating levels of GDF10. Thus, our findings identify GDF10 as an important circulating factor regulating osteogenic transdifferentiation and subsequent VC of VSMCs. Further studies are needed to establish whether patients with CKD may benefit from increased circulating levels of this protein.

## Experimental procedures

### Animal models

*TDAG51*^*−/−*^ mice were generated as previously described (64) and backcrossed to C57BL/6J (*TDAG51*^+/+^) mice for at least 9 generations. *GDF10*^*−/−*^ mice were generously provided by Dr Se-Jin Lee (65) and were also backcrossed onto the C57BL/6J background for at least 9 generations. Wild-type (C57BL/6J) mice were purchased from Jackson Laboratory (Stock No: 000664). All mice were housed in a 12-h light-dark cycle with free access to a regular control diet. For studies examining VC using a VD_3_ model of hyperphosphatemia-induced rapid VC, 8-week-old male *GDF10*^*−/−*^ mice and *GDF10*^*+/+*^ littermate controls were used. Mice were subcutaneously injected with 5.55 × 10^5^ IU/kg/day of active VD_3_ (Sigma-Aldrich) for 3 consecutive days (66) and were sacrificed 4 days posttreatment. To examine the effect of GDF10 on VC, wild-type mice were subcutaneously implanted with an osmotic pump (Alzet) with a 100 μl reservoir volume and a 1-week release duration filled with 25 μg of rhGDF10 (R&D Systems) dissolved in 4 mM HCl. The control pump was filled with 4 mM HCl. An infusion pump was implanted 3 days prior to VD_3_ injections and mice were sacrificed 4 days following the VD_3_ treatment. All animal procedures were approved by the McMaster University Animal Research Ethics Board.

### VSMC and *ex vivo* aortic ring cultures

All VSMCs were isolated from the aortas of 6 to 10-week-old male mice by the explant method (67) and cultured in Dulbecco’s Modified Eagle’s Medium (GIBCO GlutaMAX 10567-014 + 1 g/L D-glucose + 110 mg/ml Sodium Pyruvate) supplemented with 10% v/v fetal bovine serum (FBS) (Sigma-Aldrich), 100 IU/ml penicillin and 100 μg/ml streptomycin (Gibco). Primary human aortic VSMCs were purchased from Lonza (CC-2571) and cultured in SmGM-2 BulletKit medium (Lonza). Cells were plated at a confluence of 50 to 70% and used at passages 4 to 8. Aortic rings for *ex vivo* studies were isolated similarly. Briefly, thoracic aortas were removed and washed in PBS-antibiotic-antimycotic solution (Gibco) 6 times while the adventitia and adipose layers were carefully removed. Clean aortas were cut into 5 to 10 mm segments (∼4-5 per animal) and cultured in suspension (68). All VSMCs and *ex vivo* aortic rings were maintained at 5% CO_2_ at 37 °C. To induce osteogenic transdifferentiation and calcification of the VSMCs, the medium was supplemented with additional P_i_ to achieve a concentration of 2.6 mM. Previous reports have utilized varying treatment concentrations of GDF10. Thus, for the purpose of our studies, the addition of cleaved or full-length (FL) rhGDF10 (R&D #1543-BP and AbNova #1619540) at increasing doses (100-1000 ng/ml) was also tested. Similarly, varying concentrations of BMP3 (R&D #113BP; 100-200 ng/ml) were added to the calcification medium.

### Calcium quantification assay

After the decalcification of cells or tissue with 0.6 N HCl for 48 to 72 h, calcium deposition was quantified using a colorimetric calcium kit using the o-cresolphthalein method according to the manufacturer’s instructions (Sigma-Aldrich). Measurements were normalized to protein content or dry tissue weight.

### OsteoImage HA crystal quantification assay

HA deposition was quantified using the OsteoImage fluorescent assay kit (Lonza) according to the manufacturer’s instructions. Briefly, cells were plated in a black clear bottom 96-well plate and cultured in calcification media for 5 to 6 days. Cells were washed, fixed with 3.7% w/v paraformaldehyde (PFA), and stained with a fluorescent reagent. HA deposition was quantitatively assessed using a fluorescent microplate reader (Molecular Devices) at 492/520 nm wavelengths (34).

### Immunoblotting

Immunoblot analysis was performed as previously described (69). Briefly, whole cell and tissue lysates were prepared using RIPA lysis buffer containing a protease inhibitor cocktail (Roche). Protein concentrations were determined using a modified Lowry assay (Bio-Rad). Equal amount of protein samples were resolved by SDS-PAGE and subsequently transferred to nitrocellulose membranes (Bio-Rad). The membranes were then blocked in 5% w/v skimmed milk, incubated with primary antibodies for 18 h at 4°C, followed by horseradish peroxidase (HRP)-conjugated secondary antibodies. Secondary antibodies were detected using an enhanced chemiluminescence reagent (Amersham) and exposed using a Konica Minolta X-ray film processor. Band intensity was measured by densitometry analysis using ImageLab software (Bio-Rad) and normalized to loading controls. All antibodies and working dilutions are listed in Table S1.

### IHC and histological staining

Paraffin blocks were cut into 4 μm thick sections, deparaffinized, and dehydrated in 3 changes of xylene and 100% v/v ethanol, respectively. Following endogenous peroxidase treatment, sections were then blocked in 5% v/v serum and subsequently incubated in primary antibodies for 18 h at 4 °C. Sections were then exposed to biotin-labeled secondary antibodies (Vector Laboratories). Streptavidin-labeled HRP solution (Vector Laboratories) and the developing solution (Vector Laboratories) were used to visualize the staining. Calcium deposition in mouse tissue sections was visualized using Alizarin Red S stain. Following deparaffinization, sections were stained with 2% w/v Alizarin Red (Sigma-Aldrich) solution at pH 4.2 for 2 min, dehydrated, and mounted in a synthetic mounting medium. Slides were examined using a Nikon light microscope. All antibodies and working dilutions are listed in Table S1.

### *In situ* hybridization

Glass slides containing 12 μm thick frozen sections of whole-body mice at embryonic day 12 (E12) and postnatal day 5 (p5) mice, as well as 30 tissues of adult mice were fixed using 4% formaldehyde. The [^35^S]-labeled (PerkinElmer) cRNA probes were synthesized *in vitro* and corresponded to the mouse coding region of GDF10. RNA sense and antisense probes were synthesized and tested by *in situ* hybridization as reported.

### Immunofluorescence (IF)

IF staining was performed as described previously (70). Briefly, 10 μm thick cryosections of thoracic aortas were fixed for 1 h using 4% w/v PFA, permeabilized for 15 min with 0.025% v/v Triton X-100 and subsequently blocked with 5% w/v bovine serum albumin (BSA). Slides were then incubated with primary antibody overnight at 4 °C and stained with appropriate Alexa Fluor-labeled (ThermoFisher Scientific) secondary antibodies and DAPI (Sigma-Aldrich). Slides were mounted with Permafluor (ThermoFisher Scientific) and visualized using a fluorescent microscope (EVOS-FL, ThermoFisher Scientific). All antibodies and working dilutions are listed in Table S1.

### ELISA

Commercially available ELISA kits were used to measure serum levels of mouse GDF10 (Elabscience) and BMP3 (MyBioSource) according to the manufacturer’s instructions.

### Microarray analysis

Total RNA was isolated from *TDAG51*^*+/+*^ and *TDAG51*^*−/−*^ VSMCs (n = 3 per group) using a RNA purification kit (Qiagen) according to the manufacturer’s instructions. Total RNA (300 ng) for each sample was used as input RNA in Ambion WT Expression Kit (ThermoFisher Scientific) and 2.75 μg of cDNA for each sample was fragmented and labeled according to Affymetrix WT labeling and fragmentation protocol. Mouse 1.0 ST Gene Arrays v1 arrays were hybridized for 18 h at 45 °C. Arrays were washed using GeneChip Fluidics Station P450 and were scanned with Affymetrix GeneChip Scanner 7G. The relevant data are represented as the log fold change relative to *TDAG51*^*+/+*^ VSMCs.

### siRNA transfection

To block PHLDA1 expression in human VSMCs, targeting siRNA (M-012389-01-0005) and non-targeting control siRNA (D-001210-01-05) were purchased from Dharmacon. Dharmafect 1 (GE Dharmacon) transfection reagent was used according to the manufacturer’s instructions (34).

### Quantitative real-time (qRT)-PCR

Total RNA was isolated using RNA purification kit (ThermoFisher Scientific) according to the manufacturer’s instructions. A total of 2 μg of RNA was reverse transcribed using a High-Capacity cDNA Reverse Transcription Kit (ThermoFisher Scientific). PCR amplification was performed using fast SYBR Green (Applied Biosystems). Relative transcript expression levels were calculated using the ΔΔCT method and normalized to 18S. All primer sequences are listed in Table S2.

### Human serum samples

Serum from nondiabetic patients with CKD (eGFR 8-45 ml/min) and associated severe VC, or no significant calcification as assessed by CAC score were measured for GDF10 by ELISA. The study cohort included both males and females and was not stratified by other co-morbidities or biochemical or clinical parameters (71, 72). All human studies reported in the manuscript abide by the Declaration of Helsinki principles.

### Statistical analysis

Data were analyzed using the unpaired Student *t* test or 1-way ANOVA with Tukey multiple comparison testing using Prism 6 (GraphPad). Data were not analyzed for normality and equal variance. Data are presented as the mean ± SD, with *p* < 0.05 considered significant between groups. All experiments were repeated at least 3 × . Details of biological replicates are listed in all figure legends.

## Data availability

All relevant data is presented in the main manuscript and supporting information file.

## Supporting information

This article contains supporting information.

## Conflict of interest

The authors declare that they have no conflicts of interest with the contents of this article.
