# Supplementary material for: GDF10 is a negative regulator of vascular calcification
Source: J Biol Chem. 2024 Sep 21;300(11):107805. doi: 10.1016/j.jbc.2024.107805 (PMC11541827; doi:10.1016/j.jbc.2024.107805)
Supplement: Supporting Tables and Figures [file mmc1.pdf]

Gdf10 is a negative regulator of vascular calcification

Khrystyna Platko[1], Gabriel Gyulay[1], Paul F. Lebeau[1], Melissa E. MacDonald[1], Jae Hyun Byun[1], Suleiman A. Igdoura[2,3],Rachel M. Holden[4], Anna Roubtsova[5], Nabil G. Seidah[5], Joan C. Krepinsky[1,,†], Richard C. Austi1[1,*,†]*

[1]Department of Medicine, Division of Nephrology, McMaster University, and The Research Institute of St. Joseph's Hamilton, Hamilton, Ontario, Canada. [2]Biology, McMaster University Medical Centre, Hamilton, Ontario, Canada. [3]Department of Pathology and Molecular Medicine and [4]Department of Medicine, Queen's University, Kingston, Ontario, Canada. The [5]Institut de Recherches Cliniques de Montréal (IRCM), Affiliated with Université de Montréal, Montréal, Quebec, Canada

* Authors contributed equally to this work

† **To whom correspondence should be addressed:** Dr. Richard C. Austin, 50 Charlton Ave East, Room T-3313, Hamilton, Ontario, L8N 4A6. Phone: 905-522-1155 x35175; Fax: 905-540-6589; E-mail: austinr@mcmaster.ca
Dr. Joan C. Krepinsky, 50 Charlton Ave East, Room T-3311, Hamilton, Ontario, L8N 4A6. Phone: 905-522-1155 x34991; Fax: 905-540-6589; E-mail: krepinj@mcmaster.ca

Supplemental Table 1: List of antibodies used for immunoblotting (IB), immunohistochemistry (IHC) and immunofluorescence (IF)

Target antigen	Vendor or Source	Catalog #	Working concentration	Persistent ID / URL
GAPDH	Cell Signaling Technology	5174	IB: 0.34 µg/ml	https://www.cellsignal.com/products/primary-antibodies/gapdh-d16h11-xp-rabbit-mab/5174
OCN	Abcam	ab93876	IF: 1.0 µg/ml	https://www.abcam.com/osteocalcin-antibody-ab93876.html
RUNX2	Cell Signaling Technology	8486	IB: 1.82 µg/ml	https://www.cellsignal.com/products/primary-antibodies/runx2-d1h7-rabbit-mab/8486
Msx2	Novus Biologicals	NBP1-85445	IB: 0.548 µg/ml	https://www.novusbio.com/products/msx2-antibody_nbp1-85445
TDAG51	Sant Cruz Biotechnology	SC-23866	IB: 0.4 µg/ml	https://www.scbt.com/p/tdag51-antibody-rn-6e2
GDF10	Novus Biologicals	H00002662-B01P	IB:0.5 µg/ml<br>IHC: 2 µg/ml	https://www.novusbio.com/products/bmp-3b-gdf-10-antibody_h00002662-b01p

Supplemental Table 2: List of Primers Used for Quantitative Real-Time PCR

Gene	Species	Forward	Reverse
PHLDA1	Human	GAAGATGGCCCATTCAAAAGCG	GAGGAGGCTAACACGCAGG
18S	Human	GTAACCCGTTGAACCCCATT	CCATCCAATCGGTAGTAGCG
18s	Mouse	CTTAGAGGGACAAGTGGCG	ACGCTGAGCCAGTCAGTGTA
Gdf10	Mouse	AATGATCGACCAAAAGCCTGT	CTTGCAGAATACCTCACGAGC
Runx2	Mouse	ATGCTTCATTCGCCTCACAAA	GCACTCACTGACTCGGTTGG
ALP	Mouse	CCAACTCTTTTGTGCCAGAGA	GGCTACATTGGTGTTGAGCTTTT
Msx2	Mouse	GGCAACCCTGTGCTACGAAT	CCTGGACTCTCTTTTGGGCTTTA

Supplemental figures and figure legends

Fig. S1

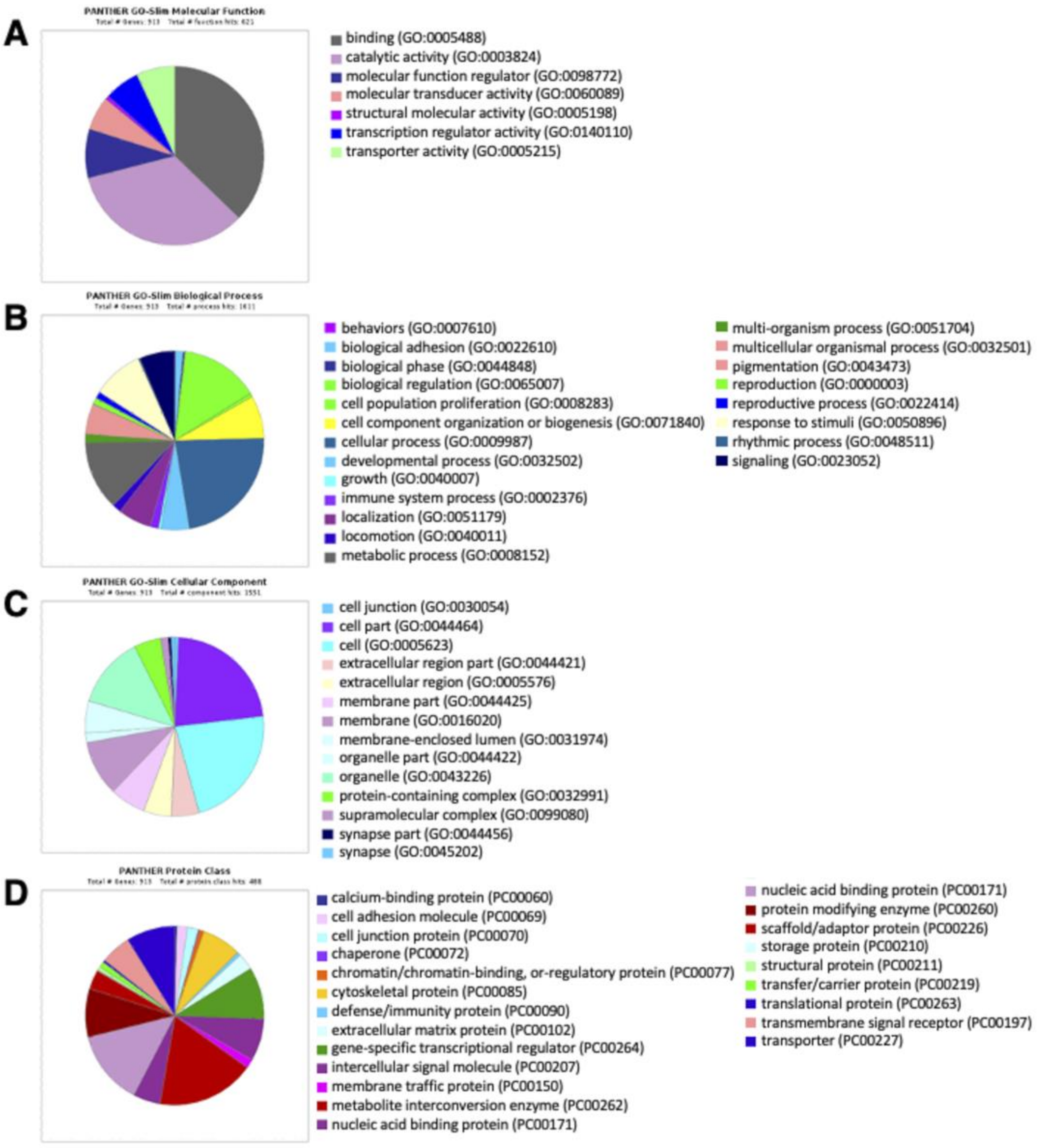

Figure S1. Gene ontology classification of targets identified by the microarray analysis. (A) Molecular function. **(B)** Biological process. **(C)** Cellular component. **(D)** Protein class.

Fig. S2

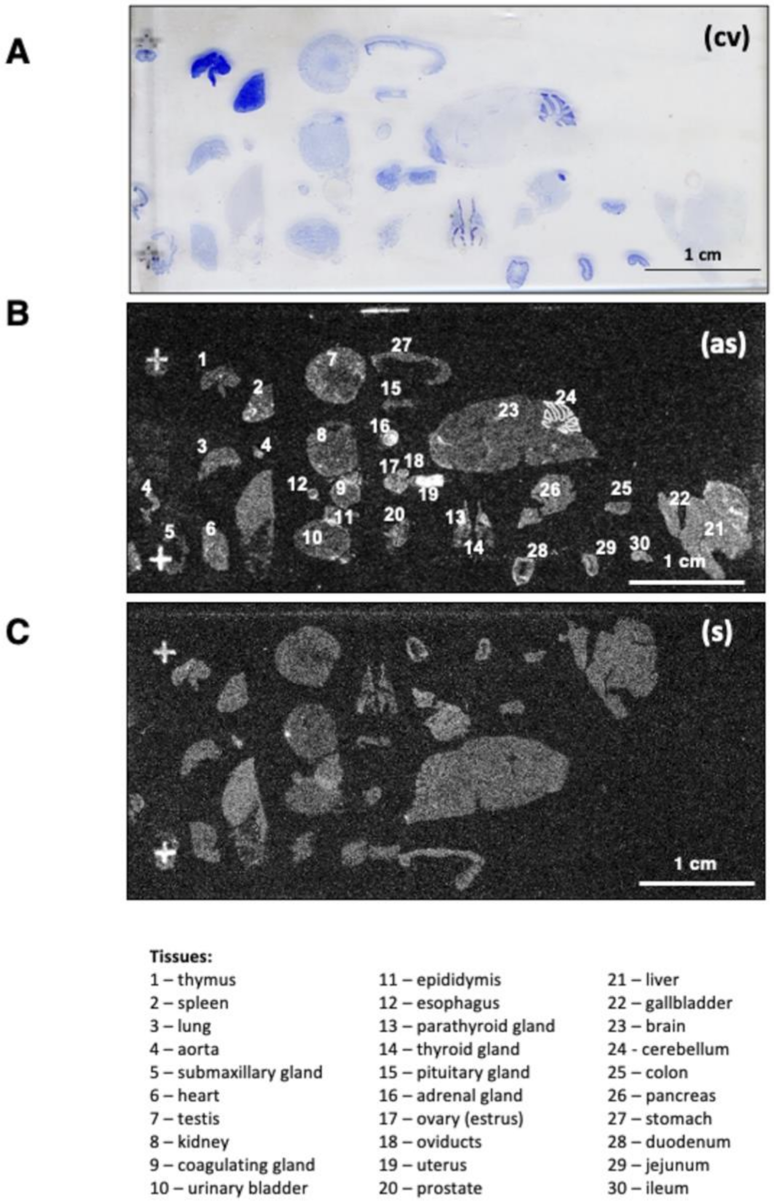

Figure S2. Local distribution of Gdf10 mRNA expression in 30 tissues of adult mice, following antisense and sense hybridization. (**A**) Frozen tissue cryostat sections in 30 mouse tissues array after thionin staining. (**B**) X-ray film autoradiography with frozen tissue cryostat sections following antisense hybridization and 5 days exposure (same slide as in A). (**C**) Control (sense) hybridization in adjacent cryostat sections showing a level of non-specific labelling (same slide as in A). As, antisense; CV, thionin staining; Gdf10, growth differentiation factor 10; S, control sense.

Fig. S3

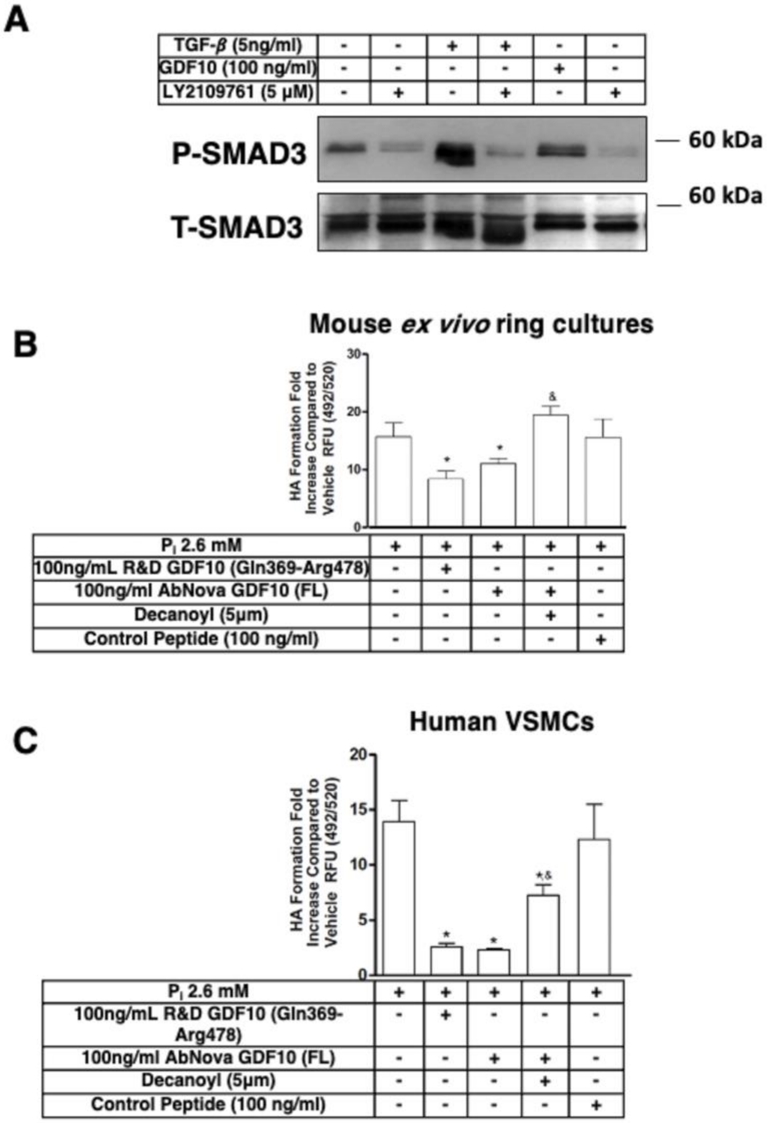

Figure S3. rhGDF10 treatment induces SMAD3 phosphorylation in TGFβ receptor-dependent manner. (A) Immunoblot analysis of P-SMAD3 and T-SMAD3 expression in VSMCs from C57BL6/J mice treated with rhGDF10 (100 ng/ml) in the presence or absence of TGFβ receptor inhibitor (LY2109761, 5 μm). Recombinant TGFβ used as a positive control (5 ng/ml). (**B** and **C**) Quantification of P_i-induced HA crystal formation and calcium deposition in mouse *ex vivo* aortic ring cultures and human VSMCs(n=8)

following 5 days of P_i (2.6 mM) treatment in the presence or absence of cleaved/active or full length rhGDF10 (100 ng/ml) as well as decanoyl (5 μm) and control peptide (100 ng/ml). All data are shown as means and error bars as S.D. *, $p<0.05$ by 1-way ANOVA with Tukey multiple comparison testing. GDF10, growth differentiation factor 10; TGFβ, transforming growth factor beta.
